# Efficacy and Safety of Therapeutic Plasma Exchange in Children with Neuroimmunological Disorders: A Limited Unicentral Study

**DOI:** 10.22037/ijcn.v18i1.40139

**Published:** 2024-03-12

**Authors:** Ali NIKKHAH, Mohammad Mahdi NASEHI, Nader MOMTAZMANESH, Kourosh ETEMAD, Somayeh HAJATNIA

**Affiliations:** 1 Pediatric Neurology Research Center, Research Institute for Children’s Health, Mofid Children Hospital, Shahid Beheshti University of Medical Sciences, Tehran, Iran.; 2 Pediatric Neurology Department, Mofid Children’s Hospital, Shahid Beheshti University of Medical Sciences, Tehran, Iran.; 3 Department of Pediatric Hematology & Oncology, Shahid Beheshti University of Medical Sciences, Tehran, Iran.; 4 Department of Epidemiology, School of Public Health and Safety & Environmental and Occupational Hazards Control Research Center, Shahid Beheshti University of Medical Sciences, Tehran, Iran.

**Keywords:** Therapeutic Plasma Exchange, Adverse Effects, Children, Pediatric, Neuroimmunological Disorders

## Abstract

**Objectives:**

Therapeutic plasma exchange (TPE) is a plasmapheresis procedure whose Safety data for pediatric neuro-immunological disorders (PNID) is confined. The present research documents TPE’s safety and feasibility data in these conditions.

**Materials & Methods:**

The current study involved six distinct groups of patients with PNID undergoing TPE: neuromyelitis optic spectrum disorder (NMOSD), autoimmune encephalitis (AIE), acute disseminated encephalomyelitis (ADEM), multiple sclerosis (MS), Guillain-Barre syndrome (GBS), and optic neuritis (ON). This study documented complications related to each TPE process. In addition, TPE’s efficacy was studied in these patients.

**Results:**

The present study recorded adverse effects in 18 patients with PNID that received 121 TPE cycles: five cycles (4.13%) in MS, three (2.48%) in AIE subgroup, one (0.82%) in ADEM, and two (1.65%) in GBS. No severe complications were observed among the patients.

**Conclusion:**

Patients with PNID tolerated therapeutic plasma exchange, which was a safe process.

## Introduction

Therapeutic plasma exchange (TPE) uses a medical instrument to split the patient’s blood and elements. The American Society declares this definition for Apheresis treatment guidelines (1). A replacement solution is used instead of the plasma. The solution can be a colloid solution such as plasma and/or albumin or a crystalloid/colloid solution mixture.

TPE is the standard treatment for neurological disorders such as myasthenia crisis and acute and chronic inflammatory demyelinating polyradiculoneuropathy (1, 2). First - line immunosuppressive treatments include intravenous immunoglobulin (IVIG), corticosteroids, and TPE in treating autoimmune encephalitis. However, the order of these therapies and the different mixtures must be better described (3-6). The present study aimed to determine the efficacy and safety of TPE in children with some common neuroimmunological disorders. Presently, data needed to select optimal therapy protocols is limited for TPE’s duration and dosage for patients with PNID (7). This reassert documented the TPE trial on kids with PNID at Mofid children’s Hospital.

## Materials & Methods

After the ethics committee approved, this study inspected a pediatric group <18 years treated with TPE at a children’s neurological center from March 2021 until April 2022. This research selected patients in PNID with multiple sclerosis (MS), neuromyelitis optic spectrum disorder (NMOSD), autoimmune encephalitis (AIE), Guillain-Barre syndrome (GBS), optic neuritis (ON), and acute disseminated encephalomyelitis (ADEM). 

The disease diagnostic standards are recorded in Table 1 (7-12). The researchers excluded patients who did not fulfill the clinical diagnostic standards (Table 1). Patients’ paraclinical and clinical conditions were studied, and data were obtained on the underlying disease, demographics (sex, age), treatment protocols, adverse events, and results. The participants expressed their informed consent before the TPE. TPE was generally applied after 3-5 days of treatment with intravenous immunoglobulin, high-dose corticosteroids, or both. In the TPE technique, the interventional radiology or surgery team inserted a double-lumen apheresis catheter under sedation. Depending on their clinical condition, patients were admitted to the pediatric intensive care unit or the pediatric neurology ward. The present study used a Spectra Optia Apheresis machine-61000 (Manufactured by TERUMO BCT, Inc., Lakewood, Colorado) for TPE procedures. Before performing TPE, this study obtained CBC, PT, PTT, INR, BUN, Cr, and electrolyte levels. Typically, the researchers exchanged plasma volume with a mixture of FFP and 5% albumin (80% to 20% ratio). All patients received prophylactically calcium gluconate (1-2 grams) during each procedure. In patients <20 kg, this research used 250 ml packed RBCs to prime the device, precluding hemodynamic instability. TPE was performed for all patients every other day for 5-10 cycles, exchanging one plasma volume for each. 

Blood laboratory values were monitored prior to each TPE process. This study tailored The TPE frequency on a daily patient evaluation basis. Common terminology criteria for adverse events’ severity (CTCAE) scored the adverse events (13). Improvement in therapy was evaluated daily based on neurological exams and caregivers’ subjective views on outcomes after initiating therapy. SPSS software (version 28) performed statistical analyses (SPSS Inc, Chicago, IL). The software analyzed the descriptive statistics and standard deviations.

## Results

**Figure 1:**
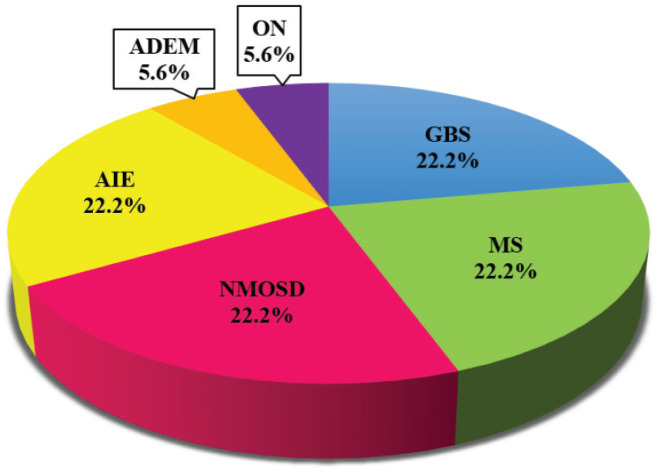
Diversity of diagnosis in patients

**Figure 2:**
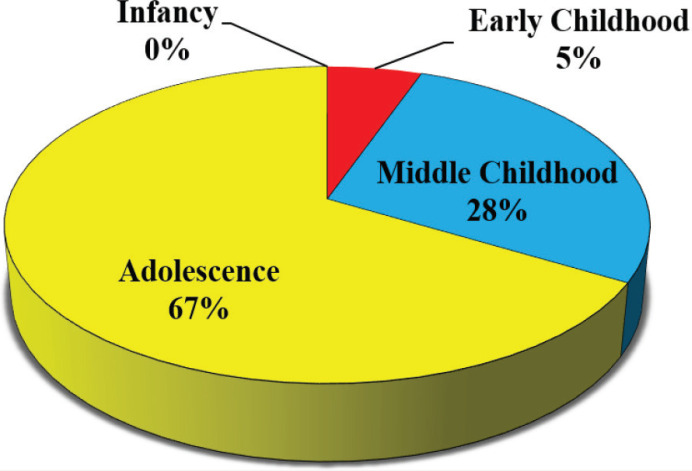
The age distribution of patients

The current study studied 18 patients who underwent 121 TPE procedure cycles (Table 2). The initial diagnosis of diseases included NMOSD in 4 (22.2%), AIE in four (22.2%), MS in four (22.2%), ADEM in one (5.6%), GBS in 4 (22.2%), and ON in one (5.6%) patients (Figure 1). The median onset age of presentation was 11.1 years, ranging from four to 15 years. Figure 2 shows the patients’ age distribution in distinct age groups. 

The female/male ratio was 1/1. Two patients in the AIE subgroup had antibodies for NMDA receptors; one was positive for voltage-gated potassium channel (VGKC), one in the NMOSD subgroup was positive for aquaporin 4, and two in the MS subgroup were OCB-positive. Prior to TPE, four patients (22.2%) received IVIG, eight (44.4%) received steroids, and five (27.7%) received both, while one patient (5.6%) did not receive any of the treatments before or after TPE. 

Patients received TPE with a combination of fresh frozen plasma (80-20%) and 5% albumin with 5-10 cycles every other day. Patients with PNID received 121 TPE cycles. The present research investigated TPE-related complications in subgroups (Table 3). Out of all the TPE cycles, three cycles (2.48%) in the AIE subgroup, two (1.65%) in GBS, five (4.13%) in MS, and one (0.82%) in ADEM, and No cycles in NMOSD and ON subgroups showed adverse effects. Grade 1 (as per CTCAE) Chill was observed in three cycles (2.48%) of MS and AIE patients, grade 1 Tachycardia in 3 cycles (2.48%) of GBS, MS, and AIE patients, grade 3 Urticaria in two cycles (1.65%) of GBS and patients with MS. Moreover, grade 2 hypertension, grade 1 dyspnea, and grade 1 Nausea were observed in one cycle (0.83%) of AIE, MS, and ADEM patients, respectively. One dose of hydrocortisone was injected IV to treat Urticaria, and Enalapril was used to control hypertension; none induced hemorrhage or infection. No mortalities were recorded during the process. 

Table 4 presents The Improving effect of TPE on neurological exams. Neurological exams comprise items of color vision (CV), visual acuity (VA), gag reflex, deep tendon reflexes (DTRs), Glasgow Coma scale (GCS), and muscle force. These exams were evaluated after a 5-10 day TPE course. Table 4 shows that in most cases, TPE significantly impacts patients with NMOSD, AIE, GBS, MS, and ADEM disorders. In contrast, TPE does not affect the visual acuity and color vision of optic neuritis patients, which may be due to the severity of damage to the optic nerve before performing the TPE.

**Table 1:** Diagnostic criteria (7-12)

AIE	All three following criteria: 1. Short-term memory loss, altered mental status, or psychiatric symptoms with rapid progression (<3 months). 2. At least one of the following: • New focal CNS findings • Seizures not explained by a previously known seizure disorder • CSF pleocytosis ( WBC>5 cell/mm3) • MRI findings indicative of encephalitis 3. Rule out other differential diagnoses
NMOSD	Diagnostic criteria for NMOSD with AQP4-IgG 1. At least one main clinical feature 2. Positive AqP4-IgG antibody 3. Rule out other differential diagnoses Diagnostic criteria for NMOSD without AQP4-IgG 1. At least two main clinical features (Optic neuritis, acute myelitis, area postrema syndrome, acute brainstem syndrome, Symptomatic narcolepsy, acute diencephalic clinical syndrome or symptomatic cerebral syndrome with NMOSD-typical brain lesions) occurring as a result of one or more clinical attacks and meeting all of the following requirements: At least one main clinical feature must be optic neuritis, acute longitudinally extensive transverse myelitis, or area postrema syndrome Dissemination in space (two or more various main clinical features) Fulfillment of additional MRI requirements, as applicable 2. Negative AQP4-IgG antibody 3. Rule out other differential diagnoses
MS	Diagnostic criteria for MS 1- Demonstration of dissemination in space by the presence of two or more T2 lesions, either: One or more periventricular One or more cortical or juxtacortical One or more infratentorial One or more in the spinal cord And either: 2- Demonstration of dissemination in time by one or more contrast-enhanced lesions and one or more non-contrast-enhanced lesions 3- CSF oligoclonal bands 4- At least one new enhanced or non-enhanced lesion in follow-up MRI 5- More than one episodes typical of multiple sclerosis (lasting>24 h, separated>30 days)
GBS	Diagnostic criteria for GBS: 1- Bilateral and flaccid weakness of limbs 2- Areflexia or marked hyporeflexia Strong supportive criteria: 1- Progression over days (usually after 2–4 weeks of plateau) 2- Relative symmetry 3- Pain at onset 4- Mild sensory symptoms or signs 5- Cranial nerve involvement 6- Autonomic dysfunction 7- Absence of fever at onset of symptoms 8- Monophasic course, time between onset to plateau 12 h to 28 days Laboratory features 1- High CSF protein level after one week of symptoms 2- CSF WBC count <50 /µl (usually <10) 3- Poor conduction or conduction block on EMG-NCV
ADEM	All required criteria: A first polyfocal, clinical central nervous system event with presumed inflammatory demyelinating cause; Encephalopathy that cannot be explained by fever; No new clinical and MRI findings emerging three months or more after the onset; Brain MRI is abnormal during the acute phase. Typically, on brain MRI: Diffuse, poorly demarcated, large (>1–2 cm) lesions involving predominantly the cerebral white matter; T1 hypointense lesions in the white matter are rare; Deep grey matter lesion can be present.
ON	Clinical criteria for ON: 1- Monocular, subacute loss of vision associated with painful eye movements, decreased contrast and color vision, and relative afferent pupillary deficit 2- Painless with all other features of (1). 3- Binocular loss of vision with all features of (1) or (2) Paraclinical criteria for ON: 1- OCT: Corresponding optic disc edema acutely or an inter-eye difference in the mGCIPL of >4% or >4μm or in the pRNFL of >5% or >5 μm within 3 months after onset 2- MRI: Contrast enhancement of the symptomatic optic nerve and sheaths acutely or an intrinsic signal increase within 3 months 3- Biomarker: AQP4, MOG, or CRMP5 antibody seropositive, or intrathecal CSF IgG (oligoclonal bands).

**Table 2:** Demographic data and outcomes of research

	AIE	NMOSD	MS	GBS	ADEM	ON
Sex, male, n (%)	3(75%)	1(25%)	1(25%)	3(75%)	1(100%)	0(0%)
Age in yrs., average (±SD)	12.5(1.12)	9.5(1.5)	12.5(1.12)	8.75(3.19)	15(0)	12(0)
Wight in kg, average (±SD)	45.25(8.53)	30.75(8.01)	53.25(13.46)	27(13.87)	50(0)	47(0)
Autoantibodies, n (%)	3(75%)+	1(25%)+	2(50%)+	-	-	-
Storied, n (%)	2(50%)	1(25%)	4(100%)	0(0%)	0(0%)	1(100%)
IVIG, n (%)	1(25%)	0(0%)	0(0%)	3(75%)	0(0%)	0(0%)
Storied and IVIG, n (%)	1(25%)	2(50%)	0(0%)	1(25%)	1(100%)	0(0%)
TPE, number, n	4	4	4	4	1	1
TPE, cycles, n	30	26	28	24	8	5
TPE, complications, n (%)*	3(2.48%)	-	5(4.13%)	2(1.65%)	1(0.83%)	-
TPE, interruption due to complications	None	None	None	None	None	None

*Based on total cycles of TPE

**Table 3:** Adverse effects of TPE in different subgroups of patients

Type of Disease	Total Procedures	Type of Complications	Number of cycle and Total Percentage (%)
MS	28	Urticaria	1 (0.83%)
		Dyspnea	1 (0.83%)
		Tachycardia	1 (0.83%)
		Chill	2 (1.65%)
NMOSD	26	-	0 (0%)
GBS	24	Urticaria	1 (0.83%)
		Tachycardia	1 (0.83%)
AE	30	Tachycardia	1 (0.83%)
		Chill	1 (0.83%)
		Hypertension	1 (0.83%)
ADEM	8	Nausea	1 (0.83%)
ON	5	-	0 (0%)

**Table 4:** The Improving impact of TPE on neurological examinations

Type of Disease	V.A	C.V	DTRs	Gag Reflex	Muscle Force	G.C.S
AIE	N.I.	N.I.	rise to 100%	W.N.	N.P.	rise to 100%
NMOSD	rise to 90%	rise to 100%	rise to 100%	rise to 100%	rise to 60%	rise to 86%
MS	rise to 70%	rise to 100%	rise to 100%	W.N.	rise to 80%	W.N.
GBS	N.I.	N.I.	rise to 100%	rise to 100%	rise to 80%	N.I.
ADEM	N.I.	N.I.	rise to 100%	W.N.	rise to 100%	rise to 100%
ON	N.E	N.E	W.N.	W.N.	W.N.	W.N.

N.I: This examination is not important for evaluating this disease.

W.N: This item was normal before TPE.

N.P: It is not possible to evaluate this item.

N.E: No effect was observed

## Discussion

In this descriptive study, the association of TPE with complications in PNID was minimal. In neurological illnesses, autoimmunity plays a critical part; thus, TPE is applied for blood cleansing from antibodies and immunologically active materials (14). Some clinics use TPE when corticosteroids and IVIG fail to affect appropriately, whereas others select to apply TPE and steroids courses concurrently in severe cases (15, 16). Limited studies compare IVIG and TPE; nevertheless, they are parallel and efficacious as therapy options. Some factors are fundamental in choosing between TPE and IVIG, such as clinician preference, convenience, and understanding of the risks and safeties of TPE. Performing TPE after IVIG results in IVIG removal being administered (8). Therefore, TPE should be done prior to IVIG. 

Nevertheless, it is widely accepted that applying TPE in pediatric patients is more problematic since technical problems are more frequent with lower blood volume, vascular access, poorer cooperation in children, and higher adverse events (17). The present study indicates that TPE is exceptionally practicable as a first-line therapy for patients with PNID if performed with professional technicians, collaboration, and proper procedure. Typical complications, such as fluidelectrolyte imbalance, fever, chills, and hypotension, have been reported; however, they can be treated instantly, are not lifethreatening, and are scarcely severe enough to stop therapy (18). Other severe complications are rare, such as vascular access problems, thrombosis, pneumothorax, bleeding due to anticoagulants, infection, and increased infection risk (19). During the TPE process in the present study, just two cycles (1.65% of all procedures) had adverse effects on grades 2 and 3, which were temporary and curable. No severe complications were observed, and TPE was generally well tolerated. In total, TPE complications in children are 2.2% to 11.0% of all techniques (20–23). The percentage of adverse effects was less frequent in this study than in similar retrospective TPE studies in pediatric neuroimmunological disorders (24, 25).

The professional surgery team and ultrasound guidance reduced the rate of catheter-related complications. TPE filtration techniques have more adverse effects than TPE centrifugation techniques (26). Furthermore, as was noted previously (27), TPE using a filtration technique resulted in almost double the number of adverse effects than TPE with the centrifugation technique. In our center, we employ the centrifugation technique, decreasing adverse effects. When albumin was used without FFP as a replacement, hypotension was more common than with FFP. In two studies conducted by Noland and Greenberg (28) and Afzali et al. (29), 5% albumin and normal saline were used as replacement fluids, and FFP was not used. In these two studies, hypotension was observed among the side effects of this treatment method. In this study, patients received a combination of FFP and 5% albumin throughout cycles. A patient developed tachycardia, hypertension, and chill simultaneously with symptoms of autonomic dysfunction during their disease. Therefore, it is challenging to understand if the hypotension was due to disease pathology or TPE. The most problematic cycles with several adverse effects, including chill and urticaria, were for a 73 kg 14-year-old girl in the MS subgroup, presumably because of being overweight (BMI = 26.8 kg/m2 -94% at risk). Furthermore, it can be related to a higher plasma dose, more plasma usage, and more susceptibility to allergy. The findings indicated that patients with MS experienced the most incidence of complications.

The study should be considered within the appropriate clinical context due to its retrospective design and limited sample size. Notably, during all TPE processes, we registered thorough data consisting of the therapy type, anticoagulants and lines, the use of FFP, the fluid type used for replacement, and complications per cycle.

## In Conclusion

The findings of this investigation demonstrate that the TPE centrifugation technique is a practical and safe procedure in the pediatric age group. Additionally, the current study showed that a professional and experienced team could apply TPE as an efficacious therapeutic intervention. This method has minimal adverse effects on pediatric neuroimmunological disorders, even if used as the initial therapy. Complications are usually negligible and either easily correctable or self-limiting. 

## Authors’ Contribution

Study conception and design: Ali Nikkhah and Mohammad Mahdi Nasehi; Data collection: Somayeh Hajatnia; Analysis and interpretation of results: Somayeh Hajatnia, Nader Momtazmanesh, and Kourosh Etemad; Drafting the manuscript: Ali Nikkhah and Somayeh Hajatnia. All authors reviewed the results and approved the final version of the manuscript.

## Conflict of Interest

The authors have no conflicts of interest to declare.
